# A plan for accelerated action on obesity

**DOI:** 10.1016/S2214-109X(23)00257-7

**Published:** 2023-07-18

**Authors:** Francesco Branca, Pavel Ursu, Victor Aguayo

**Affiliations:** a Nutrition and Food Safety, WHO, Geneva, Switzerland; b Delivery for Impact, WHO, Geneva, Switzerland; c Nutrition and Child Development, UNICEF, New York, USA

Halting the rise in obesity among children, adolescents, and adults is essential to combat the growing burden of non-communicable diseases and to improve nutrition, health, and wellbeing for all. Failing to do so threatens progress towards reducing premature mortality from non-communicable diseases by 30%—a key target of the 2030 Sustainable Development Goals.

No country is on track to achieve the global nutrition target to stop the rise in overweight and obesity among children under 5 years of age, nor to achieve the global non-communicable disease target to stop obesity among adolescents and adults. The cost of obesity and obesity-related diseases continues to rise, reaching US$990 billion per year, which accounts for more than 13% of all health-care expenditures. If no further action is taken, the economic effects from overweight and obesity are projected to cost the global economy 3·29% of gross domestic product by 2060.^1^

The obesity epidemic is one of the greatest public health and nutrition challenges of our time. The Lancet Summit on Childhood Obesity: Consequences Across the Life Course, held in March, 2023, highlighted how the burden of obesity, specifically among children, has increased substantially over the past 50 years, resulting in lifelong consequences for children and families that can be passed on to the next generation. The Summit also provided the opportunity to showcase what works in policy and planning to tackle the social, commercial, and environmental determinants of childhood obesity. With only a few notable exceptions, policy efforts to address the drivers of obesity have fallen short, thwarted by food-and-beverage industry interference in public policy making, and unhelpful narratives that blame children and families and perpetuate stigma. The Summit was an opportunity to bring together more than 2000 members of a multisectoral community around this pressing public health issue, from paediatricians and nutrition specialists, to urban planners and child rights activists.

In May, 2022, the 75th World Health Assembly (WHA 75) endorsed new recommendations for the prevention and management of obesity over the life course. It also endorsed the Acceleration Plan to STOP Obesity,^2^ which clarifies how WHO, in partnership with UNICEF, will support Member States in implementing these recommendations on the basis of individual country needs and priorities, including reporting mechanisms.

Pursuant to the endorsement of the WHA 75 recommendations, the Acceleration Plan to STOP Obesity has been designed to stimulate country-led action, with a focus on delivering for impact against the obesity epidemic between now and 2030 through five workstreams: first, priority implementation of evidence-based, cost-effective interventions, such as front-of-pack nutrition labels, restrictions to the marketing of unhealthy foods and beverages, fiscal measures to reduce the consumption of ultra-processed foods and beverages, and services for the prevention and management of obesity health care; second, tailored country-specific road maps developed according to WHO's Delivery for Impact Approach,^3^ which offers a principled and structured framework for implementation, leveraging analytics, the use of targets and acceleration scenarios, and implementation and problem-solving tools to facilitate monitoring, course correction, and reprogramming if and when needed; third, strong political and scientific advocacy at global, regional, and country levels; fourth, strong and sustained engagement with partners; and fifth, accountability and reporting to monitor implementation of the Acceleration Plan at national, regional, and global level towards 2030 targets.

Since WHA 75, 28 countries across the six WHO regions have committed to being frontrunners in leading country actions, while intercountry dialogues have taken place in four out of six regions (figure). WHO and UNICEF have worked through their global, regional, and country teams to support these frontrunner countries to accelerate their progress by designing tailored country roadmaps with clearly identified priority interventions tracked across mid-term (2025) and long-term (2030) targets, with a clear pathway towards implementation. Inter-country dialogues have also established a living platform for uniting countries and other stakeholders around a common vision for the response to the obesity epidemic that is focused on prioritising selected areas of intervention and accelerating impact at country level. The perspectives of civil society organisations and families affected by and living with obesity have been amplified and reflected throughout the process, and have proven crucial to the progress made thus far.

**Figure:**
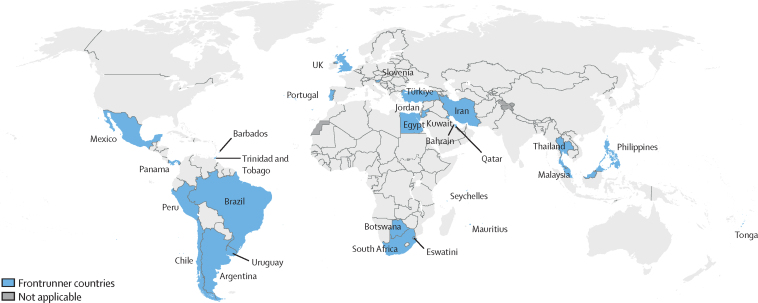
Map of frontrunner countries, as of May 2023 Frontrunner countries currently include Argentina, Bahrain, Barbados, Botswana, Brazil, Chile, Egypt, Eswatini, Iran, Jordan, Kuwait, Malaysia, Mauritius, Mexico, Panama, Peru, Philippines, Portugal, Qatar, Seychelles, Slovenia, South Africa, Thailand, Tonga, Trinidad and Tobago, Türkiye, the UK, and Uruguay.^4^ Disclaimer: the designations employed and the presentation of the material in this publication do not imply the expression of any opinion whatsoever on the part of WHO concerning the legal status of any country, territory, city or area or of its authorities, or concerning the delimitation of its frontiers or boundaries. Dotted and dashed lines on maps represent approximate border lines for which there may not yet be full agreement.

1 year following its endorsement at WHA 75, the Acceleration Plan is maturing towards the execution phase, with frontrunner countries leading the scale-up of national priority actions. All Member States are invited to accelerate action to stop obesity, drawing inspiration and experience from the frontrunner countries to inform their efforts. At WHA 76, Member States, led by Bahrain, hosted a ministerial event to announce the commencement of implementation, while highlighting the need to raise awareness about the obesity epidemic as one of the greatest public health challenges of our time. The event was also an opportunity to advocate for political commitment, increased resources, and the engagement of society at large. WHO and UNICEF will report on country progress and challenges in rolling out the Acceleration Plan in the short, medium, and long term, and on a yearly basis to the Members States through the WHA and Regional Committees platforms.

Obesity is a major public health challenge; preventing and managing obesity requires political courage and accelerated action via a united, prioritised, coherent, and adequately resourced response led by countries, with the support of other stakeholders. The uptake, scale-up, and expansion of selected interventions will be key to accelerate and sustain impact. With strong political commitment and accountable implementation, we can bend the obesity curve, and make 2030 a healthier and more sustainable year for all.

We declare no competing interests.
